# Evaluation of phytotoxicity of three organic amendments to collard greens using the seed germination bioassay

**DOI:** 10.1007/s11356-018-3928-4

**Published:** 2019-01-04

**Authors:** Tesfamichael H. Kebrom, Selamawit Woldesenbet, Haimanote K. Bayabil, Monique Garcia, Ming Gao, Peter Ampim, Ripendra Awal, Ali Fares

**Affiliations:** 1 Cooperative Agricultural Research Center, College of Agriculture and Human Sciences, Prairie View A&M University, Prairie View, TX 77446 USA; 2 Agricultural and Biological Engineering, Tropical Research and Education Center, Institute of Food and Agricultural Sciences, University of Florida, Homestead, FL 33031 USA; 3 Department of Biology, College of Science and Arts, Prairie View A&M University, Prairie View, TX 77446 USA

**Keywords:** Agricultural waste, Municipal waste, Manure, Organic fertilizer, Phytotoxicity, Nickel, Germination, Collard greens

## Abstract

Small-scale vegetable and fruit crop producers in the USA use locally available commercial organic fertilizers and soil amendments recycled from municipal and agricultural wastes. Organic soil amendments provide crops with their nutrient needs and maintain soil health by modifying its physical, chemical, and biological properties. However, organic soil amendments might add unwanted elements such as toxic heavy metals or salts, which might inhibit crop growth and reduce yield. Therefore, the objective of this study was to evaluate phytotoxicity of three commercial organic amendments, chicken manure, milorganite, and dairy manure, to collard greens using the seed germination bioassay and chemical analysis of the organic amendments. The seed germination bioassay was conducted by incubating collard greens seeds to germinate in 1:10 (*w*/*v*) organic amendment aqueous extracts. Results of this work identified phytotoxic effects of chicken manure and milorganite, but not dairy manure, to collard greens. Potentially phytotoxic chemicals such as copper, zinc, nickel, and salts were also higher in chicken manure and milorganite compared to dairy manure. In particular, nickel in chicken manure and milorganite aqueous extracts was 28-fold and 21-fold, respectively, higher than previously reported toxic levels to wheat seedlings. The results demonstrate the need for more research on phytotoxicity of commercial organic soil amendments to ensure their safe use in vegetable and fruit crop production systems.

## Introduction

Small-scale vegetable and fruit crop growers are transitioning from conventional farming to environmentally friendly and more profitable semi-organic or organic farming (Reganold and Wachter 2016); therefore, they are shifting from using conventional inorganic fertilizers to organic fertilizers and soil amendments prepared from municipal, industrial, and farm wastes (Wortman et al. 2017). Organic amendments are mixed with topsoil to improve the organic matter contents of agricultural soils and maintain soil health by modifying its physical, chemical, and biological properties (Eden et al. 2017; Ferreras et al. 2006). Organic fertilizers and soil amendments improve soil fertility, nutrient availability, soil aeration, and water holding capacity (Diacono and Montemurro 2010; Wortman et al. 2017). Interestingly, improvement in water holding capacity of soils treated with organic amendments increased the yield of maize and soybean under drought conditions (Lotter et al. 2003).

Unlike conventional inorganic fertilizers, the nutrient content and chemical composition of organic amendments prepared from agricultural and municipal solid wastes could vary depending on their origin and technologies used to convert these bioresources into organic fertilizers or soil amendments (Emino and Warman 2004; Tognetti et al. 2005). In addition, the level of nitrogen, the most limiting plant nutrient, in organic amendments is lower than in conventional inorganic fertilizers (Rigby and Smith 2014). Moreover, the nitrogen in organic fertilizers and soil amendments is slowly released to plants (Cogger et al. 2011). Therefore, to meet nitrogen requirements of crops, organic amendments are applied at higher rates (Cogger et al. 2011). Besides nitrogen, organic amendments contain plant macro- and micronutrients including phosphorus, potassium, copper, zinc, iron, and heavy metals, such as chromium, arsenic, selenium, nickel, and salts (Alvarenga et al. 2015). With higher rates of application, some of the nutrients, heavy metals or salts in organic fertilizers and soil amendments, could reach toxic levels, inhibit crop growth, and reduce yield. Therefore, determining phytotoxicity of organic soil amendments prior to their use in agricultural soils is critical for developing sustainable small-scale semi-organic or organic crop production practices that depend on bioresources recycled from municipal, industrial, and farm wastes.

In the USA, commercial organic fertilizers and soil amendments recycled from municipal and agricultural wastes are commonly used for growing small-scale vegetable and fruit crops and ornamental plants. It is also economically feasible to use commercial organic fertilizers and soil amendments as they are easily obtained from local stores (Araji et al. 2001). Furthermore, commercial organic fertilizers and soil amendments are ready for use and can be directly applied to agricultural soils without the need for further processing. However, their potential phytotoxic effects should be investigated in order to optimize their use in the production of horticultural crops and ornamental plants.

Phytotoxicity of organic soil amendments is evaluated using various chemical and biological approaches (Barral and Paradelo 2011; Luo et al. 2018). Potentially phytotoxic substances and their levels could be identified using expensive and laborious analytical chemistry methods. However, the sensitivity of plant species to phytotoxic chemicals in organic amendments may vary. Phytotoxicity of organic soil amendments could be also due to synergetic effects when the level of each of potentially toxic chemicals is lower than it can induce toxicity (Barral and Paradelo 2011; Emino and Warman 2004). Therefore, it may not be easy to identify phytotoxicity exclusively using analytical methods. In contrast, however, less expensive and quicker bioassays (e.g., seed germination test), in conjunction with chemical analysis, could be effective in determining phytotoxicity of organic soil amendments. Germination test can be conducted by incubating seeds in aqueous extracts of organic amendments or growing plants in organic amendments with or without mixing with soils (Barral and Paradelo 2011; Emino and Warman 2004; Luo et al. 2018). Such bioassays identified phytotoxicity of soil amendments prepared from plant residues, livestock farms, and biosolids from municipal and industrial wastes.

The seed germination bioassays were also used to monitor the stability and maturity of composts (Hase and Kawamura 2012; Tiquia 2010; Young et al. 2016). During composting, bacteria degrade the organic matter in the composted material. A compost is characterized as stable when it is relatively more resistant to further decomposition and mature when the phytotoxic compounds produced during the decomposition process such as ammonia disappear (Bernal et al. 2009; Komilis 2015). Stable and mature composts are applied to agricultural soils. However, heavy metals and salts from the original composted materials that are not eliminated during composting could be toxic to plants. Although commercial organic soil amendments could be stable and mature, they may contain phytotoxic substances from the original material that adversely affect crop growth and reduce yield. Therefore, we investigated the phytotoxicity of three commercial organic soil amendments commonly used in the USA (chicken manure, milorganite, and dairy manure) using a seed germination bioassay.

A widely used germination test protocol for identifying phytotoxicity is incubation of seeds to germinate in 1:10 (*w*/*v*) compost aqueous extracts (reviewed by Luo et al. 2018). Either monocot or eudicot seeds can be used for the germination test (Barral and Paradelo 2011; Luo et al. 2018). Therefore, we conducted seed germination bioassay by incubating collard greens (*Brassica oleracea* L. Acephala group) seeds in aqueous extracts of chicken manure, milorganite, and dairy manure. Furthermore, we analyzed the chemical properties and composition of the aqueous extracts. Our studies identified phytotoxicity of chicken manure and milorganite to collard greens possibly due to higher levels or synergetic effects of micronutrients, salts, and heavy metals.

## Materials and methods

### Seed germination bioassay

Phytotoxicity of commercial organic fertilizers and soil amendments was investigated in Petri dishes by incubating collard greens seeds with aqueous extracts of milorganite (Milorganite), granulated and dehydrated chicken manure (Medina), and composted dairy manure (Black Kow). Milorganite (Milwaukee Organic Nitrogen) is prepared from heat-dried microbes that digested municipal and industrial wastes from Milwaukee Metropolitan Sewerage District (MMSD) (Archer 2007). Milorganite can be used as a soil amendment and fertilizer for the production of diverse crops (Cogger et al. 2011). According to the label on the packaging materials, the nitrogen contents of milorganite, chicken manure, and cow manure is 6%, 3%, and 0.5%, respectively, which were validated in our lab by analyzing samples from these organic amendments using inductive coupled plasma optical emission spectrometer (Agilent ICP-5100) working with both radial and axial view and C, H, N, and S content using elementar vario Macro tube.

To conduct seed germination bioassay, fresh aqueous extracts (1st extract) were prepared by shaking 1 g of milorganite, chicken manure, or dairy manure with 10 ml of deionized water (1:10 *w*/*v*) on a rotary shaker for 1 h. The solutions were centrifuged at 5000*g* for 15 min, and then filtered through 0.8-μm membrane filter. A range of dilutions of the aqueous extracts using deionized water as diluent was prepared. Commercial collard greens seed variety Tiger (F1) was obtained from a seed company (Johnny’s Selected Seeds). Ten collard greens seeds were set to germinate on Whatman filter paper wetted with 4 ml aqueous extracts or deionized water (control) in 100-mm-diameter and 25-mm-height Petri dishes (Fisher Scientific) incubated at 25 °C. After 3 days, the number of germinated seeds and the radicle length of each germinated seed were documented. A seed with a radicle length of at least 2 mm was considered as germinated. A second round of aqueous extracts (2nd extract) were prepared by adding 10 ml deionized water to the residues from the first aqueous extract preparations (1st extract), incubated for 1 h on a rotary shaker, centrifuged at 5000*g* for 15 min, and then filtered through 0.8-μm membrane filter. Germination tests were conducted by incubating ten collard greens seeds with a range of dilutions of the second aqueous extracts. After 3 days, the number of germinated seeds with radicle length at least 2 mm and the radicle length of each germinated seed were documented. The whole experiment including preparation of fresh aqueous extracts and germination test was repeated four times, and the results were analyzed by determining the relative seed germination (RSG), relative radicle growth (RRG), and germination index (GI) as shown below:

$$ \mathrm{RSG}=\frac{\mathrm{Number}\ \mathrm{of}\ \mathrm{germinated}\ \mathrm{seeds}\ \mathrm{in}\ \mathrm{aqueous}\ \mathrm{extract}}{\mathrm{Number}\ \mathrm{of}\ \mathrm{germinated}\ \mathrm{seeds}\ \mathrm{in}\ \mathrm{deionized}\ \mathrm{water}\ \left(\mathrm{control}\right)}\times 100\% $$

$$ \mathrm{RRG}=\frac{\mathrm{Radicle}\ \mathrm{length}\ \mathrm{of}\ \mathrm{germinated}\ \mathrm{seeds}\ \mathrm{in}\ \mathrm{aqueous}\ \mathrm{extracts}}{\mathrm{Radicle}\ \mathrm{length}\ \mathrm{of}\ \mathrm{germinated}\ \mathrm{seeds}\ \mathrm{in}\ \mathrm{deionized}\ \mathrm{water}\ \left(\mathrm{control}\right)}\times 100\% $$

$$ G\mathrm{I}=\mathrm{RSG}\times \mathrm{RRG}\times 100\% $$

### Chemical analysis of organic fertilizers and soil amendments

The chemical properties and composition of the first and second aqueous extracts of chicken manure, milorganite, and dairy manure were analyzed using pH meter (accumet AB15), electrical conductivity (EC) meter (Milwaukee MW 802), and an ICP-OES. Heavy metals and plant macro- and micronutrients analyzed include arsenic (As), chromium (Cr), cadmium (Cd), mercury (Hg), copper (Cu), nickel (Ni), lead (Pb), selenium (Se), zinc (Zn), boron (B), iron (Fe), sodium (Na), and phosphorus (P). Calibration standards for the ICP-OES analysis were prepared from single elements (P, Na, Fe, and B) (Sigma) and pre-mixed elements (As, Cd, Cr, Cu, Mn, Ni, Hg, Pb, Se, and Zn) (Agilent) diluted with 1% HNO_3_. All reagents were analytical grade or better. The analysis was conducted on three independently prepared first and second aqueous extracts of chicken manure, milorganite, and dairy manure as described above.

### Effect of sodium salt on the germination and radicle growth of collard greens seeds

The effect of sodium salts on the germination and radicle growth of collard greens seeds was investigated using sodium chloride (NaCl) solution. A 20,000 ppm Na^**+**^ stock solution was prepared by dissolving 2.54 g NaCl in 50 ml deionized water. The amount of Na^**+**^ in the stock solution was confirmed using ICP-OES. A range of NaCl dilutions were prepared from the stock solution containing from 50 to 10,000 ppm Na^**+**^ in deionized water to a final volume of 4 ml and used in germination test in Petri dishes using the same method as described for the aqueous extracts of chicken manure, milorganite, and dairy manure.

### Statistical analysis

The number of germinated seeds and the average radicle length in each of the four biological replicates were determined. The RSG, RRG, and GI of each biological replicate were calculated using the equations shown in the “Seed germination bioassay” section of the materials and methods. The results reported in this paper are mean GI, RSG, and RRG of the four biological replicates, and the error bars are standard error of the mean (SE) calculated using the equation SE = SD/√*N*, where SD is standard deviation of the means, and *N* is the number of biological replicates. The physical properties and chemical analysis results reported in this paper were also analyzed similarly.

## Results

### Germination of collard greens in chicken manure, milorganite, and dairy manure aqueous extracts

**Fig. 1:**
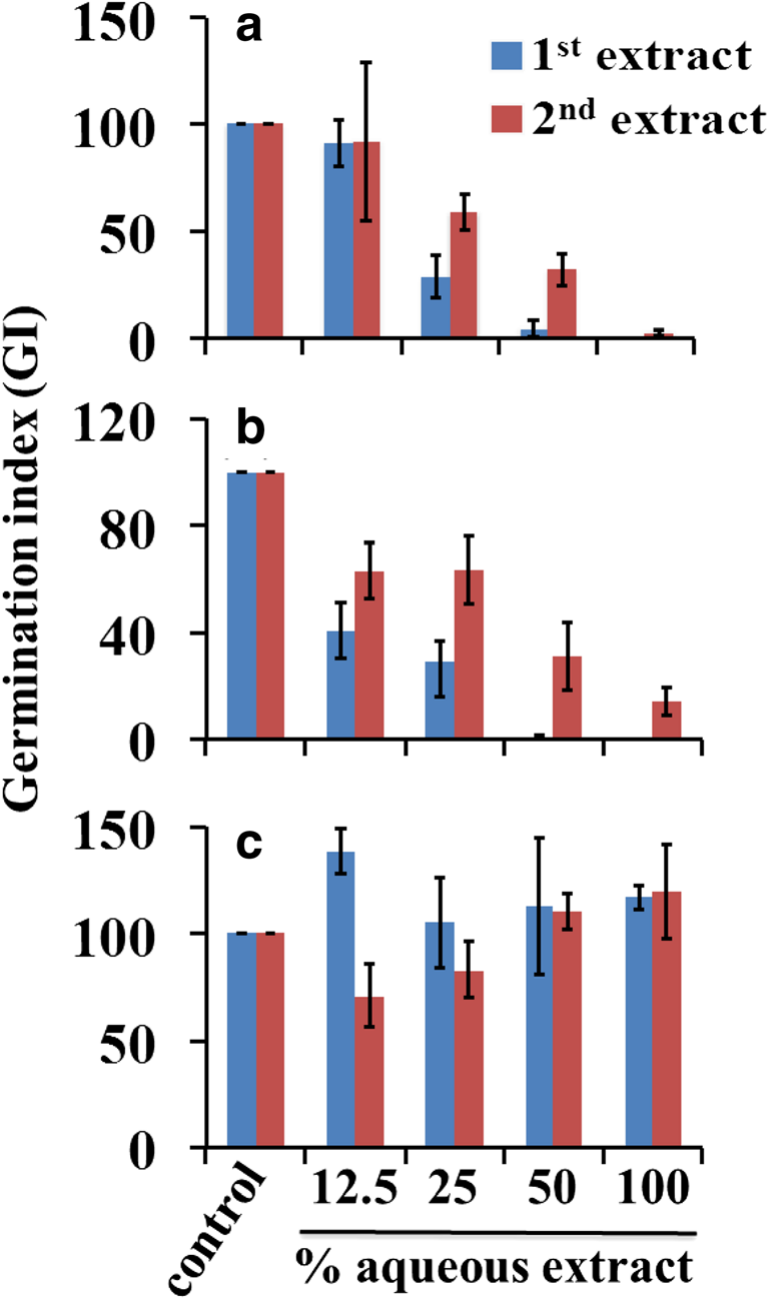
Germinated index of collard greens seeds germinated in aqueous extracts of chicken manure (**a**), milorganite (**b**), and dairy manure (**c**). GI was calculated from four independent experiments. Data are mean ± SE, *N* = 4

The seed germination bioassay identified potential phytotoxic effects of chicken manure and milorganite, but not dairy manure, to collard greens. As shown in Fig. 1, the germination index (GI) in 100% chicken manure and milorganite first aqueous extracts was 1.9% and 0%, respectively. The results indicate strong phytotoxicity of chicken manure and milorganite to collard greens. We hypothesized that the observed phytotoxicity could be due to soluble substances in the original material or phytotoxic by-products during the conversion of poultry farm and municipal wastes into chicken manure and milorganite, respectively, that could be washed away in the first extract. Therefore, we conducted germination test by incubating collard greens seeds in an aqueous extract (2nd extract) prepared by adding 10 ml of deionized water to residue from the first aqueous extract preparations. The GI in 100% chicken manure and milorganite second aqueous extracts was 2.6% and 14.3%, respectively (Fig. 1a and b). The results indicate the phytotoxic substances in chicken manure and milorganite might not be easily removed by leaching. The GI at 12.5% dilution in the first extract of chicken manure was not different from the control and significantly reduced in 25% aqueous extract compared to control (Fig. 1a). The results indicate the phytotoxic substances act in a concentration dependent manner. The GI in milorganite at 12.5% dilution was about 40.5% indicating phytotoxic effects of milorganite even at low dilution rates (Fig. 1b). Dairy manure appears to be free of phytotoxic effects (Fig. 1c). In fact, at 12.5% dilution, the GI in the first extract was 139% of the control indicating a stimulating effect of dairy manure at lower application rates (Fig. 1c). Interestingly, the GI at 12.5% dilution in the second extract of dairy manure was 71% of the control indicating mild phytotoxic effect. The GI in the 25% dilution was reduced from 105% of the control in the first extract to 83% of the control in the second extract. However, there was no difference in the GI between the first extract and the second extract when the seeds were incubated to germinate in 50% and 100% aqueous extracts. It appears that at lower dilution rates in the second extract, the stimulating effect of dairy manure disappeared resulting in mild phytotoxicity.

**Fig. 2:**
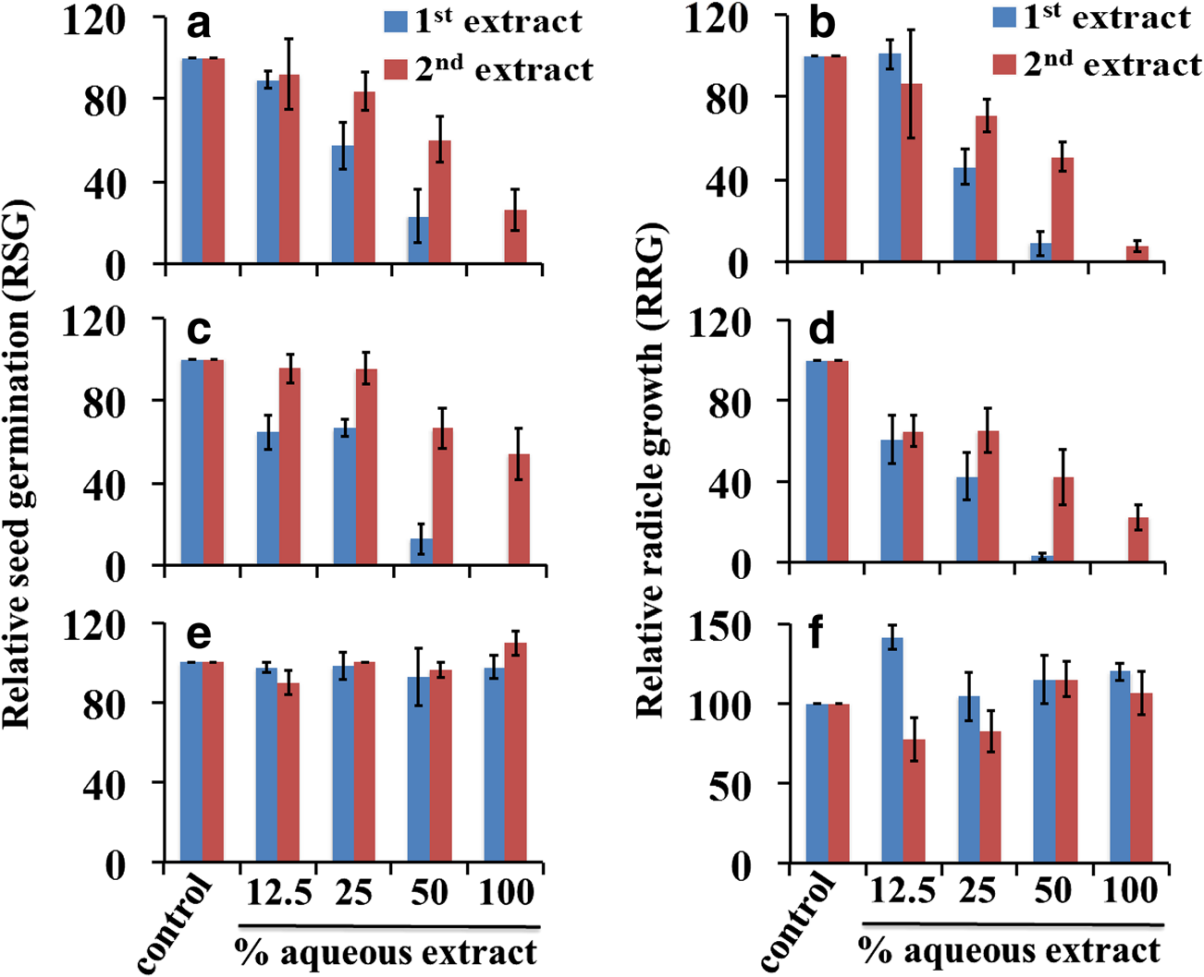
Relative seed germination (RSG) and relative radicle (root) growth (RRG) of collard greens in aqueous extracts of chicken manure (**a** and **b**), milorganite (**c** and **d**), and dairy manure (**e** and **f**). Data are mean ± SE, *N* = 4

In addition to GI, we analyzed the relative seed germination (RSG) and relative radicle growth (RRG) to see the effect of chicken manure, milorganite, and dairy manure aqueous extracts specifically on seed germination and root growth of collard greens. Both RSG and RRG were improved in 100% chicken manure and milorganite second aqueous extracts compared to the level in the first aqueous extracts, with more improvement in RSG than RRG (Fig. 2). The RSG in dairy manure first and second aqueous extracts in all dilutions were similar and comparable to the RSG in the control (Fig. 2e). The RRG in 25%, 50%, and 100% dairy manure first and second aqueous extracts were also comparable to the RRG in the control. Whereas the RRG in 12.5% dairy manure first aqueous extract was higher than the RRG in second aqueous extract and also in the control (Fig. 2f). Therefore, the stimulation of GI in 12.5% dairy manure first aqueous extract (Fig.1) was exclusively due to an increase in RRG.

### Chemical properties and composition of aqueous extracts of chicken manure, milorganite, and dairy manure

To identify phytotoxic factors in chicken manure and milorganite, we analyzed the chemical properties and composition of the aqueous extracts used in the seed germination bioassay. As shown in Table 1, the pH in both chicken manure and dairy manure was comparable and alkaline, and slightly increased in the second extract compared to the first extract. However, seed germination and radicle growth were inhibited in chicken manure aqueous extracts, but not in dairy manure aqueous extracts. The pH of milorganite aqueous extracts was 6.2 and 6.7 in the first extract and second extract, respectively. Differential response of collard greens seed germination under similar pH as in chicken manure and dairy manure and an increase in GI in milorganite from the first extract (0%) to the second extract (14%) with little change in pH indicate phytotoxicity of chicken manure and milorganite is not related to their pH.

**Table 1:** Electrical conductivity (EC) and pH of the first and second aqueous extracts of chicken manure, milorganite, and dairy manure. Data are mean ± SD; *N* = 3 independently prepared aqueous extracts

	pH		EC (mS/cm)	
	1st extract	2nd extract	1st extract	2nd extract
Chicken manure	8.0 + 0.09	8.3 + 0.51	8.1 + 0.14	2.9 + 0.12
Milorganite	6.2 + 0.04	6.7 + 0.16	2.1 + 0.06	0.6 + 0.02
Dairy manure	8.4 + 0.19	8.8 + 0.36	0.4 + 0.01	0.1 + 0.01

Electrical conductivity measurements are used to determine the level of salts in soil (He et al. 2012). A higher electrical conductivity indicates higher level of salts in a solution (Visconti et al. 2010). The electrical conductivity of the first extract of chicken manure was 8.1 mS/cm and decreased to 2.9 mS/cm in the second aqueous extract (Table 1). In milorganite, the EC in the first extract was about 2.3 mS/cm and reduced to about 0.6 mS/cm in the second extract, and in dairy manure, the EC was reduced from 0.4 mS/cm in the first extract to 0.1 mS/cm in the second extract. Although the EC in the second aqueous extracts of chicken manure and milorganite was reduced significantly, the GI in 100% aqueous extracts improved only slightly (Fig. 1).

**Fig. 3:**
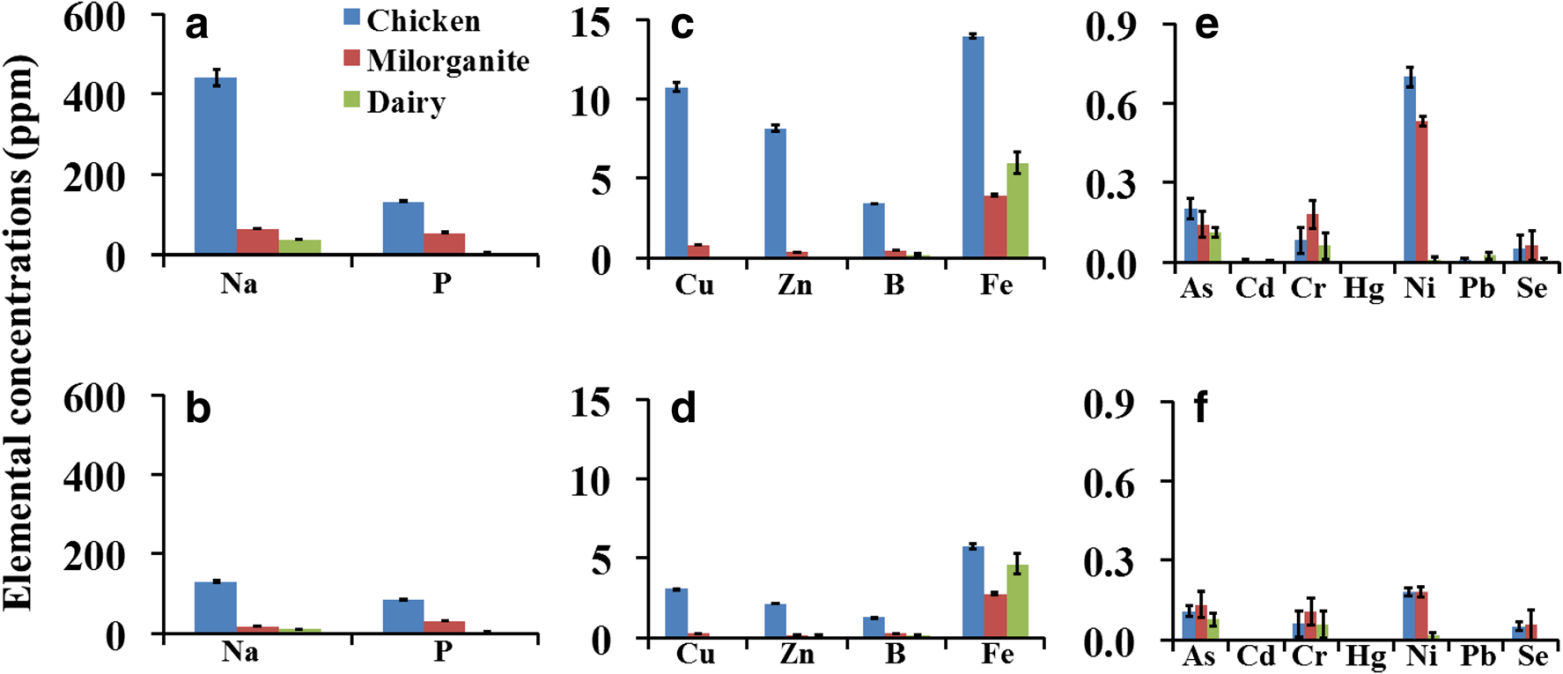
Elemental composition in the first aqueous extracts (upper panel: **a**, **c**, **e**) and second aqueous extracts (lower panel: **b**, **d**, **f**) of chicken manure, milorganite, and dairy manure. Data are means ± SE, *N* = 3 independently prepared aqueous extracts

Chemical analysis of the aqueous extracts of chicken manure, milorganite, and dairy manure might help identify potentially phytotoxic compounds. In general, the levels of most of the elements analyzed were higher in aqueous extracts of chicken manure followed by milorganite, while dairy manure had the lowest (Fig. 3). The average sodium ion (Na^**+**^) content of the first aqueous extracts of chicken manure, milorganite, and dairy manure was 442.2 parts per million (ppm), 63 ppm, and 36.3 ppm, respectively (Fig. 3). The level of Na^+^ in the second aqueous extracts of chicken manure, milorganite, and dairy manure was 129.2 ppm, 17.2 ppm, and 10.2 ppm, respectively. The level of Na^**+**^ in the aqueous extracts was consistent with their EC values, highest in chicken manure and lowest in dairy manure (Table 1). The reduction in Na^**+**^ from the first extract to the second extract (Fig. 3a and b) was also consistent with similar patterns of reduction in EC (Table 1). However, as with EC, although the level of Na^**+**^ in the chicken manure was reduced from the first to the second extract, the GI in 100% in the second extract improved only slightly.

Plant micronutrients such as copper (Cu), zinc (Zn), and iron (Fe) were relatively higher in chicken manure than in milorganite and dairy manure aqueous extracts (Fig. 3). Fe was higher in chicken manure followed by dairy manure and milorganite. It is unlikely that the low GI in 100% chicken manure first aqueous extract is due to Fe because the higher level of Fe in dairy manure compared to the level in milorganite did not affect seed germination and root growth of collard greens. The amount of Cu, Zn, and B was relatively higher in chicken manure. The amount of heavy metals and micronutrients was significantly reduced in the second aqueous extracts (Fig. 3b, d, and f). As shown in Table 2, the percentage reduction ranged from about 25% for Cr in chicken manure to more than 70% for Cu, Zn, and Na. The patterns of reduction in milorganite were similar to those in chicken manure. Interestingly, while the level of Fe in chicken manure was reduced from 13.9 ppm in the first extract to 5.7 ppm in the second extract, the level of Fe in dairy manure was reduced from 6.0 ppm in the first extract to 4.6 ppm in the second extract. Potentially toxic heavy metals including arsenic (As), chromium (Cr), and nickel (Ni) were present in chicken manure and milorganite aqueous extracts (Fig. 3e). The higher level of Ni in chicken manure and milorganite aqueous extracts compared to dairy manure suggests that the phytotoxic effect could be due to Ni. The low level or below the detection level of heavy metals such as Hg and Pb in the organic amendments could be due to regulation on the use and disposal of such hazardous substances in agricultural and municipal wastes (Jain et al. 2005). Elements such as Cd and Pb, which were already very low in the first aqueous extract of chicken manure, reduced by about 100% or they were below the detection limit in the second extract.

**Table 2:** Percentage reduction or increase (indicated by asterisks) of heavy metals, plant macro- and micronutrients in the second aqueous extracts of chicken manure, milorganite, and dairy manure compared to the first aqueous extracts

Elements	Chicken manure	Milorganite	Dairy manure
Na	70.8	72.6	71.9
P	36.2	43.0	29.4
Cu	71.6	66.7	0.0
Zn	73.7	60.6	92.5*
B	63.1	32.5	10.6
Fe	58.9	29.4	22.4
As	46.3	4.8	31.3
Cd	100.0	0.0	100.0
Cr	25.0	39.6	5.6
Hg	0.0	0.0	0.0
Ni	74.3	65.9	25.0*
Pb	100.0	0.0	100.0
Se	0.0	8.1	100.0

### Effect of sodium salts on the germination of collard greens seeds

**Fig. 4:**
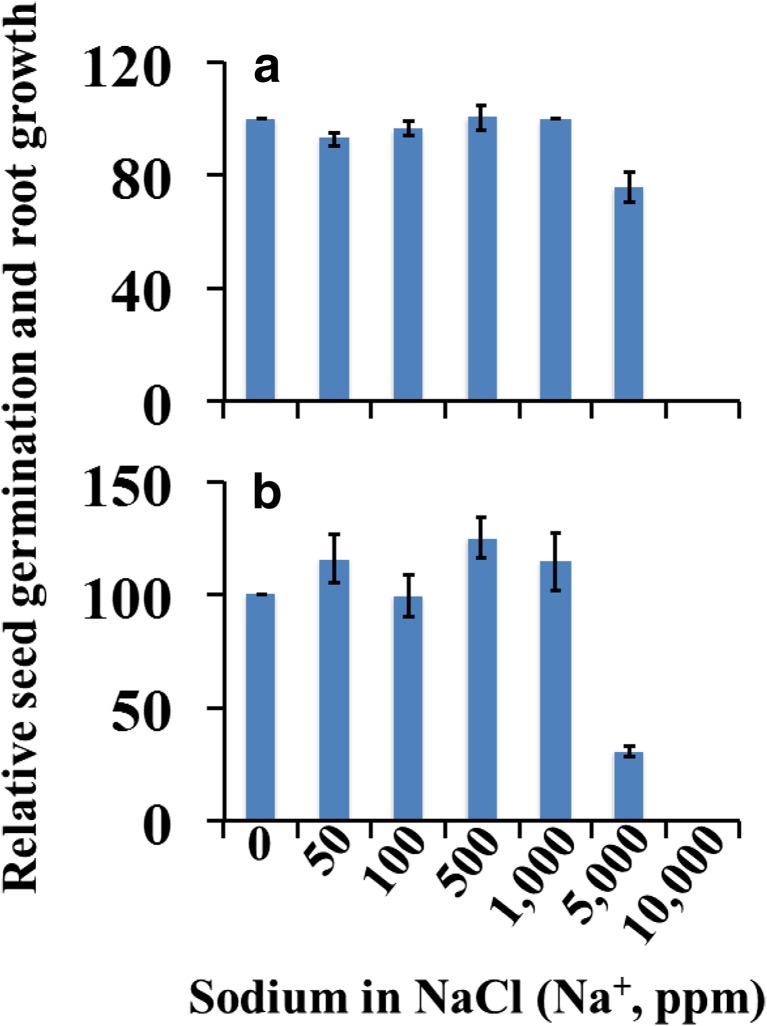
Relative seed germination (**a**) and relative radicle (root) growth (**b**) of collard greens seeds in sodium chloride solution. Data are mean ± SE, *N* = 3

Seed germination and seedling growth are inhibited in soils with high salts (Hakim et al. 2010; Hanin et al. 2016; Ibrahim 2016). It is possible that the high level of salts in chicken manure indicated by higher EC (8.1 mS/cm) and Na^+^ (442.2 ppm and 129.2 ppm in the 1st and 2nd aqueous extracts, respectively) could result in the inhibition of seed germination and radicle growth (Table 1, Fig. 3a). Therefore, we conducted germination test in a series of sodium chloride (NaCl) solutions containing from 50 to 10,000 ppm Na^**+**^ to determine the sensitivity of collard greens seed germination to sodic salts. The germination and radicle growth of collard greens seeds incubated in NaCl containing 50 to 1000 Na^+^ were not different from the control (Fig. 4). The effect of NaCl on seed germination was observed at 5000 Na^+^ ppm, in particular the radicle growth was suppressed. The germination of collard greens seeds was inhibited at 10,000 Na^+^ ppm.

## Discussion

Commercial soil amendments prepared by composting industrial, municipal, and agricultural wastes using appropriate technologies could be alternative sources of organic fertilizers by organic or semi-organic crop growers. Regulations for organic soil amendments recycled from municipal and agricultural wastes are developed with a focus on potential hazards to human health and the environment while largely ignoring quality of organic amendments as a fertilizers and their toxicity to crop plants (Cesaro et al. 2015; Rigby et al. 2016). Therefore, phytotoxicity of commercial organic fertilizers and soil amendments should be investigated prior applying to agricultural soils to reduce risk to crops. In this study, we investigated phytotoxicity of three commercial organic fertilizers and soil amendments commonly used in the USA (chicken manure, milorganite, and dairy manure), using the seed germination bioassay and analyzing chemical properties and composition of their aqueous extracts.

Germination index (GI) is commonly used to determine phytotoxicity of compost (Luo et al. 2018). A GI greater than 80% indicates that the compost is free of phytotoxicity, whereas a GI below 80% indicates potential phytotoxicity of compost to crops and a GI below 50% indicates high phytotoxicity (Barral and Paradelo 2011). The GI of collard greens seeds incubated in 100% aqueous extracts of chicken manure and milorganite was close to zero indicating strong phytotoxicity of these organic amendments. The GI at 25% dilution of aqueous extracts of chicken manure and milorganite was less than 80%. The results confirm the strong phytotoxicity of these two organic amendments to collard greens and possibly to other crops. Leaching the soil with irrigation water or after natural rainfall after application of organic amendments containing water-soluble toxic chemicals may reduce salinity and phytotoxicity (Kuepper 2003). It is also possible that some of the composting by-products such as organic acids could be washed away following irrigation or natural rainfall. To explore if this is possible with chicken manure and milorganite, we investigated the effect of second aqueous extracts, prepared by adding deionized water to the residues from the first aqueous extract preparations, on the germination of collard greens seeds. The GI in 100% of the second extract was slightly improved, but it was still below 50% (Fig. 1). Our results suggest that the phytotoxic chemicals in chicken manure and milorganite are slowly released into the solution and might not be easily removed by leaching of the soil during irrigation operations following their application.

The phytotoxicity of an organic amendment could be due to its effect on seed germination or root growth or both (Emino and Warman 2004; Tiquia et al. 1996). Understanding the contribution of each is important in developing strategies to reduce or eliminate the effect of organic amendments on seedling establishment. For example, if the phytotoxic effect is on germination and subsequent growth is not affected, seeds could be germinated in growth media in nurseries without adding organic amendments, and then, the seedlings can be transplanted to fields with soils amended with organic fertilizers. Analysis of the seed germination and root growth results indicate that the phytotoxic effects of chicken manure and milorganite were on both seed germination and root growth. However, root growth was more inhibited than seed germination. In fact, at 25% milorganite second aqueous extract, the seed germination was not affected while root growth was inhibited (Fig. 2c and d).

Dairy manure aqueous extracts did not affect the seed germination or root growth of collard greens indicating dairy manure could be free of toxic chemicals. Interestingly, when the seeds were incubated with 12.5% aqueous extracts of dairy manure, the GI was 139% of the control specifically due to enhanced growth of the radicle. The high GI indicates the stimulating effect of chemicals present in the dairy manure aqueous extracts (Moldes et al. 2007). However, this stimulatory effect of dairy manure was not only absent in the second extract but also the GI was less than 80%. In the second extract of dairy manure, either the stimulant was reduced or the inhibitor was increased or both. The results suggest that the balance between growth stimulants and inhibitors in the organic amendments could determine seed germination and seedling development. Therefore, identifying chemicals in organic amendments that stimulate or inhibit seed germination could help to develop ways to improve the usefulness of recycled organic amendments in agriculture. Furthermore organic amendments that do not have phytotoxic effect on seed germination can have an effect at later stages of plant growth when the amount of inhibitors released from the organic amendments increase. Therefore, while our results indicate that dairy manure might not be phytotoxic to collard greens and other crops, additional studies are needed to make conclusions. For example, increasing incubation and shaking time during the preparation of the aqueous extracts from 1 h used in the current experiment to 24 or 48 h or longer might be useful to confirm if dairy manure does not contain potentially phytotoxic chemicals.

Seed germination and seedling growth are sensitive to soil salinity (Bajji et al. 2002; Hakim et al. 2010; Houle et al. 2001). The sodium-ion level in chicken manure was higher than in milorganite or dairy manure, but collard greens seeds germinated at much higher levels of Na^+^. In addition, the level of sodium in chicken manure was significantly reduced from the first extract to the second extract, but phytotoxicity was only slightly reduced. It is possible that while salts in the organic amendments can contribute to their phytotoxic effects, the phytotoxicity in chicken manure and milorganite might not be exclusively due to salts as significant reduction in EC and salt from the first to the second extract did not significantly reduce or eliminate phytotoxicity.

Potentially toxic micronutrients and heavy metals identified in chicken manure and milorganite include Cu, Zn, Ni, As, and Cr. The high level of Cu and Zn in chicken manure might be due to the use of these two metals in poultry diet to protect chickens from disease and improve productivity (Bolan et al. 2010; Yazdankhah et al. 2014). Paradelo et al. (2010) identified phytotoxic effects of 10 mg/L Cu solution, which is equivalent to 10 ppm, on the germination of cress and barley. In the current study, the level of Cu in the first aqueous extracts of chicken manure was 10.8 ppm, which is comparable to the level of Cu that induced phytotoxicity in cress and barley seeds. However, the reduction in the level of Cu to 3.1 ppm in the second aqueous extract of chicken manure was not associated with elimination of phytotoxicity. The level of Zn phytotoxic to cress and barley was higher than 50 mg/L (Paradelo et al. 2010), which is sixfold higher than the level in chicken manure aqueous extracts in the current study. Therefore, it is unlikely that phytotoxicity of chicken manure could be due to Cu or Zn.

Ni is a micronutrient essential for plant growth at very low concentration, but could be toxic to germinating seeds when present at higher level (Seregin and Kozhevnikova 2006). As low as 25-μg/L nickel, which is equivalent to 0.025 ppm, in a hydroponic nutrient solution reduced the growth of wheat seedlings (Parlak 2016). The level of Ni in the first aqueous extracts of chicken manure and milorganite was 0.7 ppm (28-fold) and 0.53 ppm (21-fold), respectively, higher than the level of Ni phytotoxic to wheat seedlings as reported by Parlak (2016). Therefore, phytotoxicity of chicken manure and milorganite could be due to Ni. Interestingly, a combination of Ni and NaCl was more toxic to Indian mustard (*Brassica juncea*) growth than either Ni or NaCl on its own (Yusuf et al. 2012). Therefore, the strong phytotoxicity of chicken manure and milorganite may not be exclusively due to Ni; it can be also due to synergetic effect of Ni and several other heavy metals and salts (Barral and Paradelo 2011; Emino and Warman 2004). It is also possible that other chemicals that were not analyzed in this study could potentially be responsible for the phytotoxic effects of milorganite and chicken manure. The impact of such chemicals is likely to be even more with milorganite due to the fact that it is prepared from heat-dried microbes that digested municipal wastes (Archer 2007), and thus, organic by-products such as phenols and organic acid could be responsible for its phytotoxic effects.

## Conclusion

Commercial scale conversion of municipal and agricultural wastes into organic soil amendments provides solutions to the growing demand of semi-organic or organic farmers for organic soil amendments and appropriate management of pollutions. Commercial organic fertilizers and soil amendments might pose little concern for human health and the environment. However, using widely accepted method for determining phytotoxicity of compost to crops, we identified strong phytotoxicity of commercial chicken manure and milorganite to collard greens. We also identified a higher level of potentially phytotoxic chemicals such as Ni and sodic salts in the aqueous extracts of chicken manure and milorganite. It is possible that there may be additional phytotoxic chemicals that this study did not address. In addition, there is variation in the degree of tolerance of crops species to phytotoxic levels of micronutrients, salts, and heavy metals. Therefore, further studies of phytotoxicity of commercial organic fertilizers and soil amendments are needed for developing strategies for their safe use in semi-organic or organic vegetable and fruit crop production systems.
